# Association between Participation of Children with Disabilities and the Child, Family, and Environmental Factors in Shanghai, China: A Cross-Sectional Study

**DOI:** 10.3390/ijerph20010615

**Published:** 2022-12-29

**Authors:** Cong Xia, Qi Jing, Gang Chen, Mei Sun, Jun Lu

**Affiliations:** 1 School of Public Health, Fudan University, Shanghai 200032, China; 2 China Research Center on Disability, Fudan University, Shanghai 200032, China; 3 School of Management, Weifang Medical University, Weifang 261053, China; 4 China Rehabilitation and Health Institute, Weifang Medical University, Weifang 261053, China

**Keywords:** children, caregiver report, disability, participation, association

## Abstract

Participation is essential to a child’s health and well-being, whereas children with disabilities may lack the associated benefits because of physical restrictions. This study aims to examine the association between the participation of children with disabilities and the child, family, and environmental factors. A total of 433 children with disabilities aged 3–18 and their family caregivers were included. Three binary logistic regression models were respectively established to identify the significant factors associated with children’s home, school, and community participation. Our main empirical results showed that among 433 children with disabilities, 44.3%, 47.6%, and 58.7% of them never or seldom participated in home, school, and community activities. Child and family factors were found to be dominantly associated with children’s participation, and significant factors associated with the home, school, and community participation of children were different, including children’s disability type, sleep problems and emotional stability, caregivers’ education, mental HRQOL, rehabilitation belief, and number of children in the family. These results highlight that the participation of children with disabilities in mainland China urgently needs to be enhanced, and further research might be focused on validating the causal relationships between participation and significant child and family factors identified in this study.

## 1. Introduction

Participation is defined as “involvement in a life situation”. It plays a vital role in individuals’ physical, mental, and social development [1]. For children, participation improves muscle strength and power [2], helps develop an optimistic attitude towards life, and forms friendships [3]. However, children with disabilities experience more restrictions in their participation than typically developing children and may lack the associated benefits [4]. Moreover, low participation of children with disabilities can lead to obesity, depression, and social isolation [3,4]. Therefore, it is necessary to develop knowledge about the participation status of children with disabilities and identify the associated factors.

Prior research related to the participation of children with disabilities was centered on five main areas: (1) measurement of participation, such as the development of measurement instruments and the analysis of reliability and validity [5,6]; (2) perceptions of participation, i.e., understanding of participation from different perspectives [7,8]; (3) status of participation, which compares the participation of children with disabilities to children without disabilities [9]; (4) factors associated with participation, which explores the factors associated with the participation of children with disabilities quantitatively or qualitatively [10]; and (5) interventions of participation, i.e., developing and examining interventions to promote participation [11,12]. Recently, identifying associated factors and developing interventions to enhance participation among this population have received more attention.

Identifying associated factors may help implement targeted interventions. To date, research on the factors associated with the participation of children with disabilities has been primarily conducted in developed countries, such as the US [13,14], Canada [15,16], and some European countries [17]. Corresponding research has revealed the essential impact of environmental factors. Specifically, a Canadian study found that physical barriers limited children’s participation [16]. Availability of resources, such as rehabilitation programs, services, and information, were key determinants of community participation for children and youths with disabilities living in Canada and the United States [18]. A cross-sectional, multicenter European study of children with cerebral palsy found that participation was associated with the attitudes of peers, families, teachers, therapists, and strangers [19].

Compared to developed countries, developing countries usually have different political, economic, cultural, and social environments, and the status and factors associated with participation among children with disabilities may also be varied. In developing countries, families’ reactions to the diagnosis of disability in their children often come with denial and refusal of the diagnosis, followed by embarrassment and shame [20]. Additionally, attention is mostly paid to children’s body structures and functions, whereas activities and participation are less likely to be included [21]. Aside from social and cultural norms, inappropriate infrastructure and poor accessibility to services in these countries may also lead to the overwhelmingly negative participation of children with disabilities [18,22]. However, the corresponding research is sparse. One study from Thailand explored the participation in children with cerebral palsy via qualitative in-depth interviews and found that nonacceptance or overprotection from family, financial problems, inconvenient transportation, and limited health service were associated with low participation [22]. Similarly, a qualitative Saudi study found facilitators, including child’s physical ability, mother’s support, and barriers, including family responsibility, mother’s psychological status, and environmental constraints, to the participation of children with Down syndrome. The small body of preliminary literature conducted in developing countries suggests that both native spiritual culture and poor material environment can hinder children’s participation [20].

China has the largest absolute number of children with disabilities in the world, and the investigated number of children with disabilities in 2006 was 3.87 million, roughly 4.66% of the total population with disabilities [23]. However, to our knowledge, there has been little research on the participation among children with disabilities in mainland China. This reflects the fact that China, similar to other developing countries, pays little attention to the participation of children with disabilities. However, China’s economy has grown rapidly in recent years, and much effort has been made toward the rehabilitation and health development of children with disabilities [24,25]. Due to the unique cultural belief, economy, public policy, and other circumstances, the results of studies conducted in other countries cannot be transferred to mainland China. Further research is thus needed for more knowledge and understanding of Chinese conditions for the participation of children with disabilities. In this study, we selected Shanghai as the sample area. As an international metropolis, Shanghai’s economy is prosperous, and it is at the forefront of rehabilitation technology, assistive devices, financial assistance, and physical barrier-free construction [26]. Nevertheless, we seldom see children with disabilities on public transportation and in public places in our daily lives, which means their participation may not be promising. Therefore, under the visible changing environment of continuous improvement of the support system, it is necessary and of profound theoretical and practical implications to understand the participation of children with disabilities.

Many models and international literature have divided the factors associated with the participation of children with disabilities into three main categories: child, family, and environmental factors [27]. King and colleagues [28] also developed a conceptual model of the child, family, and environmental factors affecting the participation of children with disabilities and examined the conjunctive association. After that, many studies comprehensively mapped out the child, family, and environmental factors that supported or hindered the participation of this population. In this study, we explored factors associated with participation among children with various disabilities in Shanghai, based on the well-established framework. This exploration might provide references for further research to identify causal relationships.

## 2. Materials and Methods

### 2.1. Participants and Procedure

All participants were selected from a survey: “Research on the healthy development and social support system for children with disabilities”. It focused on the physical, mental, and social adaptation of children with disabilities aged 0–18, their utilization of rehabilitation services, and the social support systems needed. In this survey, the children’s information was reported by their primary family caregivers, considering the high dependence of children with disabilities. Caregivers were included if (1) their children were diagnosed with one or more kinds of disability (e.g., speech disability, intellectual disability); (2) their children were aged 0–18 years; (3) they were primary family caregivers who took care of children for more than 40 h per week and were familiar with the children’s daily lives, health, and rehabilitation; and (4) they consented to participate in this investigation.

In China, it is difficult to find older children in rehabilitation institutions. Hence, we collected data in both rehabilitation institutions and community-based settings. The convenience sampling method was used to select the children and their primary family caregivers from 12 rehabilitation institutions and 8 communities (*shequ*) in 8 districts of Shanghai from December 2019 to January 2020 and August to September 2020. In each investigation area, the following procedure was used to collect information. First, we contacted the staff of the institution and the community Residents’ Committee (*shequ jumin weiyuanhui*) to inform them of our survey purpose and the inclusion criteria of family caregivers; second, the staff of the institution and the community Residents’ Committee contacted the family caregivers of children with disabilities to inform them of the study purpose, to confirm their consent, and to form a survey arrangement (including the names of family caregivers and the survey time); third, according to the survey arrangement, trained investigators from our research team were arranged to visit the rehabilitation institutions and the offices of the community Residents’ Committees to conduct the survey. Before the investigation, investigators confirmed family caregivers’ informed consent again. If family caregivers agreed to participate in the study, they completed the questionnaire with the help of the investigators. Finally, 496 family caregivers of children with disabilities participated in this survey. For this study, we focused on the home, school, and community participation of children with disabilities. In mainland China, the usual school age for children is 3 years old. Therefore, to ensure that the sample size for the analysis of the three dependent variables was consistent, children aged < 3 years and their caregivers were excluded, and 433 children aged 3–18 years were the subjects proposed for analysis. Ethics approval was obtained from the Ethics Committees of the School of Public Health of Fudan University (Grant No. IRB#2019-10-0782).

### 2.2. Measures

#### 2.2.1. Outcomes: Participation

Over the last two decades, participation has received increasing attention, and many children’s participation measures have been developed (e.g., Assessment of Preschool Children’s Participation, Children’s Assessment of Participation and Enjoyment, Child and Adolescent Scale of Participation, Child Engagement in Daily Life Measure, Participation Environment Measurement for Children and Youth) [29]. These measures are available to measure the participation of children with disabilities in a fine-grained way, but there are many difficulties in using these scales in this study: (1) these scales usually include many items and involve many activities, which takes caregivers a long time to complete; (2) since the participation level of children with disabilities in mainland China may not be high, and caregivers’ awareness of participation may be weak, many of the contents are not applicable; (3) these measures have not been validated in children with disabilities in mainland China.

Participation is defined as “involvement in a life situation” [1]. Life situations, defined as sets of activity categories, were identified for three settings—home, school, and community—by previous literature [30]. Hence, we designed three questions based on literature and ICF-CY codes to estimate the children’s participation. These questions were then reviewed by the research team and expert advisors to ensure reliability and validity (Table 1). The pilot testing, which was conducted from December 2019 to January 2020, recruited 169 family caregivers, and 154 of them were included in the analysis. The Cronbach’s α was 0.885, and the exploratory factor analysis extracted one component, reflecting good reliability and validity. Among the total sample, children with disabilities were capable of participating in one or more kinds of activities. For each question, the level of participation was measured on a 5-point Likert scale, from 1 being “never” to 5 being “daily”.

#### 2.2.2. Independent Variables

Child factors (6 items): (1) age; (2) gender; (3) disability type: visual, hearing, and speech, physical, intellectual, mental, multiple, and developmental delay; (4) disability severity: mild versus severe disability; (5) sleep: suffered sleep problems (e.g., somnolence, dreaminess) during the last month (yes/no); and (6) emotion: emotional stability during the last month, rated on a 3-point scale with 1 = “unstable” and 3 = “stable”. According to the People with Disabilities Act of the People’s Republic of China, disability is divided into visual disability, hearing disability, speech disability, physical disability, intellectual disability, mental disability (including autism spectrum disorder), multiple disability, and other disabilities. Disability severity was classified into four levels, ranging from level I (most serious) to IV (least serious). Of children with mild disabilities, many were diagnosed with a disability, but the severity was unrated. In this study, mild disability referred to unrated, level III, and level IV, and severe disability referred to level I and level II.

Family factors (8 items): (1) gender of the caregiver; (2) caregiver’s relationship to the child: parent versus grandparent; (3) educational level of the caregiver: low (junior high school and below), intermediate (senior high school/technical secondary school), and high (junior college and above); and (4) health-related quality of life (HRQOL) of the caregiver: measured by a 12-item Short Form Health Survey (SF-12). The SF-12 [31] is a self-reported instrument used to assess physical HRQOL and mental HRQOL from 8 domains (physical functioning (PF), role-physical (RP), bodily pain (BP), general health (GH), energy/fatigue (VT), social functioning (SF), role-emotional (RE), and mental health (MH)). More family factors include (5) rehabilitation belief of the caregiver: caregivers’ expectations of the outcome of the child’s rehabilitation self-care/self-reliance. Rehabilitation belief was measured by asking family caregivers one question: “What are your current expectations for your child’s rehabilitation?” Four options were available to them: “a. Some degree of self-care to minimize the burden on caregivers; b. Achieving self-care and being able to live on their own in the future; c. To have the ability to learn and participate in cultural and spiritual life on the basis of self-care; d. Be self-sufficient and able to work and serve society”. Among the four options, a and b were included in self-care, c and d were included in self-reliance. The final few family factors included (6) the number of children (only one versus more than one); (7) economic status: measured by monthly per capita household income; and (8) family cohesion (disunity/unity).

Environmental factors (5 items): (1) public policy, (2) social attitude, (3) infrastructure, (4) rehabilitation service, and (5) education service. The first four items were rated on a 5-point scale with 1 = “dissatisfaction” and 5 = “satisfaction”. Education service was measured by the enrollment in compulsory education for children (yes/no).

### 2.3. Statistical Analysis

Descriptive analyses were expressed as mean ± SD for continuous variables, whereas categorical variables were described as frequencies and percentages. Home, school, and community participation of children with disabilities measured in this study were classified into two levels, low and high frequency. Three binary logistic regression models were respectively established to identify the facilitators and barriers of children’s home, school, and community participation. Scores greater than the mean score of all children were identified as “high frequency”, whereas scores lower than the mean score were identified as “low frequency”. In binary logistic regression analysis, we used home participation (low or high), school participation (low or high), or community participation (low or high) as “dependent variables”, and the independent variables used were the child, family, and environmental factors. These factors were identified according to the following procedures: first, we referred to the conceptual model of the child, family, and environmental factors affecting the participation of children with disabilities developed by King and colleagues. Then, considering the cultural differences, we modified the factors (including deletions and additions) based on both literature reviews and expert consultations. Finally, we conducted an expert validation. We examined the goodness of fit of all models with the Hosmer and Lemeshow test. Results of the regression models were presented as odds ratios (OR) and 95% confidence intervals (95%CI). *p* < 0.05 was defined as being statistically significant. All statistical analyses were performed using SPSS version 25.0 (IBM Corp., Armonk, New York, NY, USA).

## 3. Results

### 3.1. Participants

Table 2 shows the child, family, and environmental characteristics of 433 caregivers and their children. Of the 433 caregivers, 71.1% were female, 71.8% were parents, and 58.8% of them received high education. Among the 433 children with disabilities, 62.1% were boys, 24.5% were diagnosed with a physical disability, and 74.6% were mildly disabled.

### 3.2. Participation Patterns

Among 433 children with disabilities, 44.3%, 47.6%, and 58.7% of them never or seldom participated in home, school, and community activities. Totals of 67.0%, 69.7%, and 76.7% of them participated in home, school, and community activities, scoring ≤3. Merely 23.6%, 9.5%, and 11.3% of them participated daily in home, school, and community activities, respectively (Table 3).

### 3.3. Factors Associated with Participation of Children with Disabilities

Table 4 presents three models of factors influencing the home, school, and community participation of children with disabilities, respectively.

Model 1, with home participation as the dependent variable, was statistically significant (χ2 = 60.769, *p* < 0.001), explained between 13.7% (Cox and Snell R-square) and 18.4% (Nagelkerke R-square) of the variance for participation frequency, and correctly classified 65.9% of cases. Two significant factors associated with the home participation of children with disabilities were the caregiver’s mental HRQOL (OR = 1.034, 95% CI = 1.014, 1.055) and the rehabilitation belief (OR = 1.933, 95% CI = 1.163, 3.215).

Model 2, with school participation as the dependent variable, was statistically significant (χ2 = 89.731, *p* < 0.001), explained between 19.6% (Cox and Snell R-square) and 26.5% (Nagelkerke R-square) of the variance for participation frequency, and correctly classified 70.3% of cases. Children’s disability type and emotional stability, caregiver’s education level, and rehabilitation beliefs were found to be significantly associated with children’s school participation.

Model 3, with community participation as the dependent variable, was statistically significant (χ2 = 49.996, *p* < 0.01), explained between 11.5% (Cox and Snell R-square) and 15.3% (Nagelkerke R-square) of the variance for participation frequency, and correctly classified 65.5% of cases. Children without sleep problems were found to be associated with higher participation frequency in community places, such as parks and squares (OR = 1.912, 95%CI = 1.145, 3.195), whereas lower community participation frequency was associated with more than one child in the home (OR = 0.560, 95%CI = 0.352, 0.890).

The results of the 3 models indicated that child, family, and environmental factors were differentially associated with home, school, and community participation.

## 4. Discussion

Our study explored the child, family, and environmental factors associated with participation in Shanghai of children with various disabilities. Overall, our main findings were: (1) Participation of children with disabilities in mainland China was not encouraging; (2) child and family factors were found to be dominantly associated with children’s participation; and (3) heterogeneity was found in significant factors associated with home, school, and community participation among children with disabilities.

### 4.1. Participation Patterns

Among 433 children with disabilities, 44.3%, 47.6%, and 58.7% of them never or seldom participated in home, school, and community activities, respectively. This result is consistent with previous studies and validates our hypothesis that the participation of children with disabilities is not promising. Research from Serbia found that 65% of children with cerebral palsy never participated in household chores [32]. Children with disabilities spend most of their time at home. Participating in home activities reinforces children’s sense of belonging to the family and develops their ability for future independent living [33]. School participation of children with disabilities is a key indicator of children’s inclusion in an educational context and is fundamental for their skill development [34,35,36]. Additionally, rehabilitation is a well-known valuable approach for the healthy development of children with disabilities, and the ultimate goal is to integrate them into society, especially the community [37,38]. We did not use our questionnaire to investigate children in the general population and thus limit the comparison. However, we interviewed more than 20 mothers of typically developing children, and more than 90% of them reported daily participation in home, school, and community activities among their children. Therefore, the participation frequency of children with disabilities did not allow for optimism, and hence, it needs more concern.

### 4.2. Associated Factors

Overall, among the child, family, and environmental factors associated with the home, school, and community participation of children with disabilities, we found the key roles of child and family factors. The factors were differentially associated with home, school, and community participation.

Among child factors, disability type and emotional stability were found to be significantly associated with school participation. Specifically, children with a physical disability, intellectual disability, and developmental delay were found to have a lower frequency of school participation. Children’s emotional stability was found to be positively associated with school participation. Children with disabilities, especially those with autism spectrum disorder, are often recognized as having higher emotional problems [39]. Emotional–behavioral problems in children with disabilities, for example, hyperactivity and aggression, increase the risk of hurting themselves and other typically developing children [40]. In that case, children with emotional–behavioral problems may be limited to activities that are engaged in individually, due to the concern for the protection of other children [41]. Additionally, teachers may provide them with more assistance, which results in a lack of autonomy [42].

Children’s sleep problem was found to be negatively associated with community participation. This finding is not surprising and is consistent with previous research [27,43]. Sleep problems are common in children with disabilities. In our study, 26.8% of children with disabilities reported sleep problems, such as somnolence, difficulty falling asleep, night waking, and dreaminess. Richdale et al. [44] reported that 58.6% of children with an intellectual disability have a sleep problem. Previous studies have verified the broadly negative effects of sleep problems on children’s emotions, cognition, energy, and verbal skills [45,46]. All of these may limit children’s community participation, but it needs further validation.

Among family factors, we found that better mental HRQOL of caregivers was positively associated with a higher frequency of home participation among children with disabilities. As previous research pointed out, caring for a child with a disability comes with a variety of challenges, such as concerns about children’s development [47], high financial burden [48], and discordant marital relations [49]. Thus, many caregivers of children with disabilities may suffer the risk of mental disorders, such as anxiety, depression, etc. [50]. The relationship between caregivers’ mental HRQOL and children’s home participation is easy to understand: (1) caregivers with good mental HRQOL tend to be more optimistic, cope positively with adversity, and encourage children to contribute to family life [51]; (2) children who participate in home activities frequently have better physical function and may subsequently promote caregivers’ happiness [52].

The rehabilitation belief of family caregivers was found to be significantly associated with children’s home and school participation. Rehabilitation is crucial for children with disabilities, and the rehabilitation belief of family caregivers is vital for rehabilitation adherence. Caregivers with high rehabilitation beliefs, i.e., high rehabilitation expectations, expect their children to be able to make independent decisions, be socially competent, and realize self-reliance [32]. However, the rehabilitation belief of caregivers varies depending on their cognition, knowledge, attitude toward rehabilitation, children’s severity of disabilities, rehabilitation effect, and many other factors. Family caregivers with higher rehabilitation beliefs may encourage their children to participate more at home and at school, but in fact, the relationship between rehabilitation beliefs and home and school participation is difficult to explain.

In addition, we found that whether or not children with disabilities had siblings was associated with their community participation. Having brothers and sisters was related to a lower frequency of community participation among children with disabilities. The possible reason is that children with disabilities, due to their physical functional limitations, often need the companionship of a caregiver to help them get to public places, such as parks. If that is so, when they have siblings, the caregiver’s time and energy will be diverted, and subsequently, the child will have fewer opportunities to go outside. This explanation should be further validated, due to the cross-sectional design.

### 4.3. Limitation

There were some limitations in this study. First, we measured the participation of children with disabilities merely by using three simple questions to get an approximate estimate of the status. Next, future research should develop a reliable and valid measure of participation that is culturally suitable for people in mainland China. Second, there was a risk of chance findings, owing to the large number of independent variables included in the analysis. Third, this was a cross-sectional study, so it was difficult to establish a causal relationship between children’s participation and related factors. In the future, we could design a longitudinal study to find the causal links. Finally, we included a wide range of ages and all types of children with disabilities, which adds to the difficulty of making interventions “targeted”. Hence, further research should refine specific disability types and narrow the age range to increase the value of guiding practice.

## 5. Conclusions

This study provides knowledge to stakeholders about the participation status and a comprehensive perspective of the associated child, family, and environmental factors of children with disabilities. Home, school, and community participation are vital for the health and development of children with disabilities. However, our results indicate that this population in mainland China participates in the above three settings at a low frequency. In response to the current situation in mainland China, all stakeholders need to emphasize the importance of participation and take appropriate support measures to promote children’s participation together. Heterogeneity was found in significant factors affecting children’s home, school, and community participation. The findings of significant factors in this study provide references for further research to identify causal relationships to the participation of children with disabilities.

## Figures and Tables

**Table 1:** Participation settings and corresponding questions.

Settings	Questions
Home	In the last 4 weeks, how often did your child participate in the following activities at home, such as playing with toys or dress-up, listening to music, doing arts and crafts, washing the dishes, taking out the garbage, setting the table, or cleaning the room or other areas of the house?
School	In the last 4 weeks, how often did your child participate in group games in school, such as classroom discussions, tests, in-class assignments, hide and seek, or getting together with peers?
Community	In the last 4 weeks, how often did your child participate in activities in public places, such as parks, oceanariums, zoos, botanical gardens, supermarkets, or children’s playground?

**Table 2:** Characteristics of child, family, and environment (N = 433).

Characteristics		n (%)/Mean ± SD
Child	Age	7.12 ± 3.38
	Gender	
	Male	269 (62.1)
	Female	164 (37.9)
	Disability type	
	Hearing and speech	80 (18.5)
	Vision	32 (7.4)
	Physical	106 (24.5)
	Intellectual	52 (12.0)
	Mental	57 (13.2)
	Developmental delay	53 (12.2)
	Multiple	53 (12.2)
	Disability severity	
	Severe	110 (25.4)
	Mild	323 (74.6)
	Sleep problem	
	Yes	116 (26.8)
	No	317 (73.2)
	Emotion stability	2.40 ± 0.80
Family	Caregiver gender	
	Male	125 (28.9)
	Female	308 (71.1)
	Caregiver relation to child	
	Parents	311 (71.8)
	Grandparents	122 (28.2)
	Caregiver education	
	Low	79 (18.2)
	Intermediate	102 (23.6)
	High	252 (58.2)
	Caregiver physical HRQOL	51.41 ± 8.36
	Caregiver mental HRQOL	41.83 ± 12.87
	Caregiver rehabilitation belief ^a^	
	Self-care	134 (31.1)
	Self-reliance	297 (68.9)
	Number of children ^a^	
	1	298 (69.1)
	≥2	133 (30.9)
	Economic status (¥) ^b^	
	<3000	68 (15.8)
	3000–4999	105 (24.4)
	5000–9999	139 (32.3)
	≥10,000	118 (27.4)
	Family cohesion ^c^	
	Disunity	275 (63.7)
	Unity	157 (36.3)
Environment	Public policy	2.93 ± 0.97
	Social attitude	3.01 ± 0.89
	Infrastructure	3.19 ± 0.99
	Rehabilitation service	4.46 ± 0.61
	Education service ^d^	
	Yes	148 (34.7)
	No	279 (65.3)

N.B. The total percentage of some variables is not equal to 100, due to rounding. ^a^: 2 data missing; ^b^: 3 data missing; ^c^: 1 data missing; ^d^: 6 data missing.

**Table 3:** Participation of children with disabilities (N = 433).

	Never	Seldom	Sometimes	Often	Daily
	1	2	3	4	5
Home participation	112 (25.9)	80 (18.5)	98 (22.6)	41 (9.5)	102 (23.6)
School participation	172 (39.7)	82 (18.9)	48 (11.1)	90 (20.8)	41 (9.5)
Community participation	24 (5.5)	182 (42.0)	126 (29.1)	52 (12.0)	49 (11.3)

**Table 4:** Models 1–3 for factors associated with the home, school, and community participation of children with disabilities.

Variable	Model 1: Home		Model 2: School		Model 3: Community	
	OR	95%CI	OR	95%CI	OR	95%CI
Child						
Age	1.008	0.906–1.123	0.905	0.805–1.018	1.037	0.931–1.155
Female	0.914	0.586–1.427	0.778	0.487–1.243	0.887	0.573–1.372
Disability type ^#^						
Visual	0.601	0.208–1.737	1.209	0.392–3.728	0.739	0.266–2.048
Physical	0.580	0.273–1.231	0.437 *	0.205–0.933	0.485	0.236–0.999
Intellectual	0.600	0.246–1.464	0.364 *	0.143–0.929	0.959	0.405–2.269
Mental	1.107	0.464–2.639	0.474	0.196–1.148	0.814	0.354–1.870
Developmental delay	0.851	0.364–1.991	0.330 *	0.142–0.764	1.344	0.590–3.061
Multiple	0.738	0.307–1.771	0.566	0.226–1.417	0.984	0.420–2.305
Severe disabled	1.043	0.584–1.865	0.975	0.525–1.809	0.682	0.387–1.204
Without sleep problems	0.712	0.425–1.193	1.392	0.795–2.436	1.912 *	1.145–3.195
Emotion stability	1.026	0.765–1.377	1.641 *	1.181–2.279	1.127	0.840–1.512
Family						
Male caregivers	0.751	0.458–1.231	0.994	0.592–1.668	0.866	0.534–1.405
Grandparents	0.797	0.428–1.487	0.808	0.412–1.586	1.170	0.638–2.144
Education ^†^						
Intermediate	0.652	0.316–1.345	0.429 *	0.198–0.929	0.974	0.481–1.972
High	0.657	0.308–1.401	0.798	0.364–1.748	1.271	0.612–2.639
Physical HRQOL	1.025	0.994–1.057	0.984	0.952–1.017	1.022	0.991–1.054
Mental HRQOL	1.034 *	1.014–1.055	1.001	0.980–1.022	1.009	0.990–1.028
Self-reliance	1.933 *	1.163–3.215	3.198 **	1.809–5.653	0.900	0.543–1.493
More than one child	0.719	0.450–1.148	0.954	0.585–1.555	0.560*	0.352–0.890
Economic status	1.089	0.864–1.373	1.126	0.878–1.445	1.079	0.858–1.356
Harmonious family	1.450	0.895–2.350	0.844	0.511–1.394	0.816	0.509–1.308
Environment						
Social policy	1.037	0.805–1.338	1.064	0.817–1.385	0.940	0.733–1.206
Social attitude	0.975	0.734–1.294	1.267	0.948–1.693	1.067	0.809–1.407
Infrastructure	0.802	0.632–1.017	1.118	0.871–1.434	0.900	0.712–1.138
Rehabilitation service	1.315	0.896–1.929	1.019	0.680–1.526	1.356	0.926–1.986
Education service	0.598	0.291–1.226	0.520	0.240–1.125	1.809	0.891–3.674

^#^ For the children’s disability type, hearing and speech disability is Ref. ^†^ For the education of caregivers, low education is Ref. Hosmer and Lemeshow Test: Model 1 (χ2 = 2.426, *p* = 0.965); Model 2 (χ2 = 6.936, *p* = 0.544); Model 3 (χ2 = 3.569, *p* = 0.894). * *p* < 0.05; ** *p* < 0.001.

## Data Availability

The datasets used and/or analysed during the current study are available from the corresponding author on reasonable request.

## References

[1] WHO (2001). International Classification of Functioning Disability and Health (ICF).

[2] Balic M.G., Mateos E.C., Blasco C.G., Fernhall B. (2000). Physical fitness levels of physically active and sedentary adults with Down syndrome. Adapt. Phys. Act. Q..

[3] Di Marino E., Tremblay S., Khetani M., Anaby D. (2018). The effect of child, family and environmental factors on the participation of young children with disabilities. Disabil. Health J..

[4] Solish A., Perry A., Minnes P. (2010). Participation of children with and without disabilities in social, recreational and Leisure Activities. J. Appl. Res. Intellect..

[5] Arvidsson P., Dada S., Granlund M., Imms C., Bornman J., Elliott C., Huus K. (2020). Content validity and usefulness of Picture My Participation for measuring participation in children with and without intellectual disability in South Africa and Sweden. Scand. J. Occup. Ther..

[6] De Bock F., Bosle C., Graef C., Oepen J., Philippi H., Urschitz M.S. (2019). Measuring social participation in children with chronic health conditions: Validation and reference values of the child and adolescent scale of participation (CASP) in the German context. BMC Pediatr..

[7] Liao Y.T., Hwang A.W., Liao H.F., Granlund M., Kang L.J. (2019). Understanding the participation in home, school, and community activities reported by children with disabilities and their parents: A pilot study. Int. J. Environ. Res. Public Health.

[8] Vanska N., Sipari S., Haataja L. (2020). What makes participation meaningful?. Using Photo-Elicitation to interview children with disabilities. Phys. Occup. Ther. Pediatr..

[9] Chien C., Rodger S., Copley J. (2017). Differences in patterns of physical participation in recreational activities between children with and without intellectual and developmental disability. Res. Dev. Disabil..

[10] Steinhardt F., Ullenhag A., Jahnsen R., Dolva A.S. (2021). Perceived facilitators and barriers for participation in leisure activities in children with disabilities: Perspectives of children, parents and professionals. Scand. J. Occup. Ther..

[11] Ullenhag A., Granlund M., Almqvist L., Krumlinde-Sundholm L. (2020). A strength-based intervention to increase participation in leisure activities in children with neuropsychiatric disabilities: A pilot study. Occup. Ther. Int..

[12] McGuire M., Long J., Esbensen A.J., Bailes A.F. (2019). Adapted dance improves motor abilities and participation in children with Down syndrome: A pilot study. Pediatr. Phys. Ther..

[13] Rimmer J.H., Riley B., Wang E., Rauworth A., Jurkowski J. (2004). Physical activity participation among persons with disabilities-Barriers and facilitators. Am. J. Prev. Med..

[14] Rosenfeld L. (2020). Participation of children with disabilities across the life course: Using socio-ecological perspectives to guide research and practice. Dev. Med. Child Neurol..

[15] Shikako-Thomas K., Shevell M., Schmitz N., Lach L., Law M., Poulin C., Majnemer A. (2013). Determinants of participation in leisure activities among adolescents with cerebral palsy. Res. Dev. Disabil..

[16] Williams U., Law M., Hanna S., Gorter J.W. (2019). Personal, environmental, and family factors of participation among young children. Child Care Health Dev..

[17] Buffart L.M., Westendorp T., van den Berg-Emons R.J., Stam H.J., Roebroeck M.E. (2009). Perceived barriers to and facilitators of physical activity in young adults with childhood-onset physical disabilities. J. Rehabil. Med..

[18] Anaby D., Law M., Coster W., Bedell G., Khetani M., Avery L., Teplicky R. (2014). The mediating role of the environment in explaining participation of children and youth with and without disabilities across home, school, and community. Arch. Phys. Med. Rehab..

[19] Colver A., Thyen U., Arnaud C., Beckung E., Fauconnier J., Marcelli M., McManus V., Michelsen S.I., Parkes J., Parkinson K. (2012). Association between participation in life situations of children with cerebral palsy and their physical, social, and attitudinal environment: A cross-sectional multicenter European study. Arch. Phys. Med. Rehabil..

[20] Alwhaibi R.M., Aldugahishem H.M. (2019). Factors affecting participation in physical activities in Saudi children with Down syndrome: Mothers’ perspectives. Disabil. Rehabil..

[21] Tantilipikorn P., Watter P., Prasertsukdee S. (2012). Identifying assessment measures and interventions reported for Thai children with cerebral palsy using the ICF-CY framework. Disabil. Rehabil..

[22] Earde P.T., Praipruk A., Rodpradit P., Seanjumla P. (2018). Facilitators and barriers to performing activities and participation in children with cerebral palsy. Pediatr. Phys. Ther..

[23] National Bureau of Statistics (2007). Main Data Bulletin of the Second National Sample Survey on Disabled Persons in 2006 (no.2).

[24] Zhang H.X., Wang Y. (2020). Economic institutional changes, industrial structure evolution and high-quality growth of China’s economy. Reform Econ. Syst..

[25] Zhao X., Zhang C. (2018). From isolated fence to inclusive society: The transformational disability policy in China. Disabil. Soc..

[26] Xia C., Sun M., Li X., Lu C., Gao X., Lu J., Chen G. (2020). Health-related quality of life and related factors among primary caregivers of children with disabilities in Shanghai, China: A cross-sectional study. Int. J. Environ. Res. Public Health.

[27] Alghamdi M., Chiarello L.A., Palisano R., McCoy S. (2017). Understanding participation of children with cerebral palsy in family and recreational activities. Res. Dev. Disabil..

[28] King G., Lawm M., King S., Rosenbaum P., Kertoy M.K., Young N.L. (2009). A conceptual model of the factors affecting the recreation and leisure participation of children with disabilities. Phys. Occup. Ther. Pedi..

[29] Chien C., Leung C., Schoeb V., Au A. (2020). A Chinese version of the young children’s participation and environment measure: Psychometric evaluation in a Hong Kong sample. Disabil. Rehabil..

[30] Coster W., Law M., Bedell G., Khetani M., Cousins M., Teplicky R. (2012). Development of the participation and environment measures for children and youth: Conceptual basis. Disabil. Rehabil..

[31] Ware J.E., Kosinski M., Keller S.D. (1996). A 12-item short-form health survey—Construction of scales and preliminary tests of reliability and validity. Med. Care..

[32] Milićević M. (2020). Home participation of children with and without cerebral palsy in Serbia: An exploratory study. Disabil. Rehabil..

[33] Imms C., Reilly S., Carlin J., Dodd K. (2008). Diversity of participation in children with cerebral palsy. Dev. Med. Child Neurol..

[34] Şahin S., Kaya Kara Ö., Köse B., Kara K. (2020). Investigation on participation, supports and barriers of children with specific learning disabilities. Res. Dev. Disabil..

[35] Kaelin V.C., Wallace E.R., Werler M.M., Collett B.R., Rosenberg J., Khetani M.A. (2021). Caregiver perspectives on school participation among students with Craniofacial Microsomia. Am. J. Occup. Ther..

[36] Galvin J., Froude E.H., McAleer J. (2010). Children’s participation in home, school and community life after acquired brain injury. Aust. Occup. Ther. J..

[37] Arakelyan S., Maciver D., Rush R., Ohare A., Forsyth K. (2020). Community-based participation of children with and without disabilities. Dev. Med. Child Neurol..

[38] Carey H., Long T. (2012). The pediatric physical therapist’s role in promoting and measuring participation in children with disabilities. Pediatr. Phys. Ther..

[39] Shields N., Epstein A., Jacoby P., Kim R., Leonard H., Reddihough D., Whitehouse A., Murphy N., Downs J. (2020). Modifiable child and caregiver factors that influence community participation among children with Down syndrome. Disabil. Rehabil..

[40] Ghaffari S., Kalantari M., Rezaee M., Akbarzadeh Baghban A. (2020). Predictors of Leisure Participation in 6 to 14-Year-Old Children with Cerebral Palsy: Structural Equation Modeling. Iran J. Child Neurol..

[41] Bedell G., Coster W., Law M., Liljenquist K., Kao Y., Teplicky R., Anaby D., Khetani M.A. (2013). Community Participation, Supports, and Barriers of School-Age Children With and Without Disabilities. Arch. Phys. Med. Rehabil..

[42] Cho M., Rodger S., Copley J., Chien C. (2018). Participation in school-related activities that require hand use for children with and without developmental disabilities. J. Intell. Disabil. Res..

[43] King G., Law M., Hanna S., King S., Hurley P., Rosenbaum P., Kertoy M., Petrenchik T. (2006). Predictors of the leisure and recreation participation of children with physical disabilities: A structural equation modeling analysis. Child. Health Care.

[44] Richdale A., Francis A., Gavidia-Payne S., Cotton S. (2000). Stress, behaviour, and sleep problems in children with an intellectual disability. J. Intellect. Dev. Dis..

[45] Horne R.S.C., Wijayaratne P., Nixon G.M., Walter L.M. (2019). Sleep and sleep disordered breathing in children with down syndrome: Effects on behaviour, neurocognition and the cardiovascular system. Sleep Med. Rev..

[46] Kelmanson I.A. (2018). Sleep disturbances and behavioural and emotional problems in preschool children with bilateral cerebral palsy. Somnologie.

[47] Williams N.A., Villachan-Lyra P., Marvin C., Chaves E., Hollist C., Hatton-Bowers H., Barbosa L.N.F. (2021). Anxiety and depression among caregivers of young children with Congenital Zika Syndrome in Brazil. Disabil. Rehabil..

[48] Woodman A.C., Mawdsley H.P., Hauser-Cram P. (2015). Parenting stress and child behavior problems within families of children with developmental disabilities: Transactional relations across 15 years. Res. Dev. Disabil..

[49] Uskun E., Gundogar D. (2010). The levels of stress, depression and anxiety of parents of disabled children in Turkey. Disabil. Rehabil..

[50] Xia C., Zheng H.Y., Zhang S.Y., Tang L., Jing Q., Chen G., Sun M., Lu J. (2021). Modifiable personal and environmental factors associated with anxiety in family caregivers of children with disabilities: A comparison between parents and grandparents. J. Affect Disord..

[51] Blum R.W., Resnick M.D., Nelson R., Stgermaine A. (1991). Family and peer issues among adolescents with Spina-Bifida and Cerbral-Palsy. Pediatrics.

[52] Pagan R. (2020). Gender and age differences in loneliness: Evidence for people without and with disabilities. Int. J. Environ. Res. Public Health.

